# When Human–Wildlife Conflict Turns Deadly: Comparing the Situational Factors That Drive Retaliatory Leopard Killings in South Africa

**DOI:** 10.3390/ani11113281

**Published:** 2021-11-16

**Authors:** Julie S. Viollaz, Sara T. Thompson, Gohar A. Petrossian

**Affiliations:** 1 Wildlife Crime Research Officer, United Nations Office on Drugs & Crime, P.O. Box 600, Wagramer Strasse 5, A-1400 Vienna, Austria; 2 Doctoral Candidate, School of Criminal Justice, Rutgers-Newark University, 123 Washington Street, 5th, Floor Newark, NJ 07102-3026, USA; saratt@scj.rutgers.edu; 3 Associate Professor, John Jay College of Criminal Justice, 524 West Street, 59th, New York, NY 10019, USA; gpetrossian@jjay.cuny.edu

**Keywords:** human–wildlife conflict, human–leopard conflict, criminology, situational crime prevention, illegal killings, retaliatory killings, rational choice theory

## Abstract

**Simple Summary:**

To better understand why retaliatory leopard killings caused by human-wildlife conflict happen in rural farming communities in South Africa and how to prevent them, this study interviewed conservationists, officials, and farmers living in a small village in the Western Cape Province. The respondents described four main problems that led to these killings: (1) the government’s response to the problem of human-leopard conflict is slow and unwilling; (2) this response was not effective; (3) there were inadequate resources to correctly respond to these killings; and (4) there was a lack of laws and their application as well as strong distrust between everyone involved, making it even harder to deal with the problem. Local community members had various innovative ideas that can be implemented to better handle the problem of human-leopard conflict in their region, which are highlighted in this article. Coupled with the criminological techniques proposed in this research, the problem of human-leopard conflict can be significantly reduced with local ideas and resources, in both the region and in other parts of the world that suffer from similar problems.

**Abstract:**

Retaliatory killings caused by human-wildlife conflict have a significant impact on the survival of leopards. This study explores the reasons for retaliatory killings of leopards by interviewing community members in a small village in South Africa that experienced high incidences of human–leopard conflict. The semi-structured interviews focused on the reasons why retaliatory leopard killings occurred and how to best mitigate the situational factors that triggered these killings. Respondents cited four main problems that fueled these killings: the government’s response to human–leopard conflict was slow and unwilling; this response involved inefficient methods; there were inadequate resources to respond to these killings; and there was a clear lack of laws or their application. Local stakeholders provided a range of innovative strategies to reduce human-leopard conflict and retaliatory killings. While all parties expressed different reasons why these solutions were or were not effective, their conclusions were often similar. The distrust that existed between the parties prevented them from recognizing or accepting their common ground. Based on existing human–wildlife conflict mitigation techniques and solutions identified by local stakeholders, this article explores how criminological techniques, including situational crime prevention, can help identify and frame effective interventions to reduce the number of illegal leopard killings driven by human-wildlife conflict.

## 1. Introduction

Illegal killing of wildlife is a global threat to species conservation [1,2,3]. From small animals to megafauna, countless species are affected by illegal killing. These animals can be killed by various easily accessible tools, such as firearms, traps, pitfalls, nets, and poisons [4,5,6]. Some species are illegally killed for consumption of their meat or for the products they can produce, such as skins, shells, tusks, and horns [7,8,9,10]. These products can be used for medicinal, ornamental, and cultural reasons [11,12,13,14,15]. Animals can also be killed as a response to human-wildlife conflict, which stems from multiple reasons, such as high human population density, competition for resources due to habitat encroachment and disturbance, destruction of crops, predator-game conflicts, and predator–livestock conflicts [2,16,17,18,19,20,21]. Predation on livestock is the most common type of human-wildlife conflict [22,23,24]. Livestock predation can cause severe threats to the livelihoods of villagers, especially in certain parts of Africa, Asia, and South America [16,25,26,27].

Retaliatory killings are a reactionary response from wildlife attacks on livestock or people or destruction of crops. Human-wildlife conflict, and as a result, retaliatory killings, occur globally and affect multiple species, including carnivores and, more specifically, leopards [28,29,30]. Due to habitat encroachment by humans, some leopards are forced out of their land [31]. If a leopard is unable to move to another habitat due to limited availability of wild land and protected areas, the animal will revert to hunting alternative prey including livestock [32,33,34]. Most of the carnivorous species that fall victim to retaliatory killings prey or are suspected of preying on livestock [35,36,37]. 

A species that is directly impacted by human–wildlife conflict and retaliatory killings is leopards [28,38]. The illegal killing of leopards occurs throughout their range in mid- to southern–Africa, parts of the Middle East, and Asia [16,39,40]. In some locations, leopard killings are opportunistic and are clustered in areas with human-wildlife conflicts between farmers or villagers and leopards [41]. Leopards can move to other habitats to avoid human disturbance; however, this disturbance often involves habitat fragmentation, which leaves these animals vulnerable to extinction through other stressors like territoriality issues with other animals, illness, inbreeding, environmental disasters, etc. [31,42,43,44]. Human–leopard conflict is especially prevalent in South Africa, and leopard populations are already at risk of extinction in the Eastern and Western Cape Provinces because of habitat fragmentation and resulting lack of genetic diversity [25,33,45,46]. However, leopards have a positive impact on the ecosystem by performing many functions, such as population control through predation [47]. For these reasons, it is important to preserve leopard populations by mitigating human-leopard conflict to reduce the number of retaliatory killings.

Environmental criminology, the framework adopted for this research, has successfully been applied to wildlife crime, for example, to understand and prevent parrot poaching, illegal, unreported, and unregulated fishing, and wildlife poaching, using the techniques of situational crime prevention [48,49,50,51,52,53]. All these studies examine the illegal killing of wildlife and propose specific solutions to address such criminal activity through understanding the circumstances that facilitate wildlife crime and the use of situational crime prevention techniques to address it. Environmental criminology refers to a family of theories (the rational choice perspective, choice-structuring properties, and situational crime prevention) that “share a common interest in criminal events and the immediate circumstances in which they occur” [54] (p. 1). These theories focus on explaining the immediate circumstances that make the commission of crime possible rather than the criminal [55]. As such, their overarching goals are to (a) analyze the patterns of crime; (b) understand the crime event; and subsequently, through this understanding of the crime patterns and events, (c) prevent and control crime through specific techniques that modify the risk-benefit analysis that offenders make when choosing to commit a crime [54]. 

This qualitative study adds to the body of environmental criminology literature on wildlife crime by exploring the reasons why retaliatory leopard killings occur in a small village in South Africa. It also applies the situational crime prevention framework to make recommendations on mitigation and prevention strategies that can be locally implemented to address retaliatory leopard killings more effectively. 

## 2. Materials and Methods

The interviews for this study took place in the Western Cape Province of South Africa, near a small farming community, with the exception of one interview which was conducted at a private nature reserve, two and a half hours east of the community. Most of the surrounding land is primarily fynbos habitat, divided into meat and dairy livestock farms, with the rest of the land designated as crop and game farms. Most landowners are white and speak English, while farm hands tend to be black and speak Afrikaans. The authors picked the area because it was experiencing significant human-leopard conflict, did not attract much tourist revenue, and lacked government oversight. Reports of retaliatory leopard killings were plentiful at the time and, as such, it was an ideal location to study tolerance of leopard killing [56].

A total of 16 participants living in or near the community were interviewed during July and August 2011: seven (7) livestock farmers, three (3) conservation non-governmental organization (NGO) staff, and six (6) government officials. A local NGO provided the names of farmers and government officials to interview, and the researcher used snowball sampling to identify additional participants. They recommended a combination of farmers who applied leopard-friendly livestock farming techniques and farmers who opposed their conservation work as even those who opposed were eager to share their point of view. In one such case, a farmer took the researcher on his cattle rounds to show her his daily routine. The conservationist practitioners interviewed came from local NGOs, private wildlife reserves, and the Western Cape Province government wildlife service, Cape Nature, and two other related departments (The Department of Economic Development, Tourism, and Environmental Affair for Free State Province, and the Tourism and Parks Agency, Mpumalanga Province) in Free State and Mpumalanga Provinces. Given the small, close-knit nature of the community, being vouched for by a community member allowed farmers to share leopard problems more freely with the researcher and made snowball sampling necessary.

The researcher contacted the recommended individuals by phone and asked if they would be willing to talk about “leopard poaching” in the region; all, but one person, agreed. Emphasis was placed on the fact that the research was not affiliated with any local actor, respondents’ identities would be kept confidential, and the interview was meant to be a fact-finding mission to understand why leopards were being killed in the area and to benefit from respondents’ insider knowledge of the situation. The overarching goal of this research was to understand the problem through a crime prevention lens by focusing on the (rational) choices made by those who illegally killed leopards and to design prevention interventions.

The semi-structured interviews with local residents and conservation practitioners lasted about two hours, with the researcher visiting respondents at their homes after calling to set up an appointment. By this time, the researcher had been residing in the area for two months and was, therefore, tangentially known to the community. Although a list of interview questions was drawn up prior to the interviews, the flow of conversation determined the topics covered with occasional prompts based on the questions (see Appendix B for the list of pre-prepared interview questions). All respondents were told they could stop the interview and withdraw their consent to participate at any time, as well as skip any questions they did not wish to answer. Only cursory notes were taken during interviews to put respondents at ease and consisted of a few key words to jog recall. Conversations were typed up from memory, based on the key words written, immediately following the interview. Two of the government officials were unavailable in person and answered questions via email, with that correspondence taking place between April and September 2011, and were added to the in-person interview data.

A content analysis of the interview data was conducted in Atlas.ti using a series of codes, generated inductively, to identify major themes respondents discussed and attitudes they held as to why retaliatory leopard killings occur in the study site and what contextual factors triggered them. An inductive method limited preconceptions about the data, allowing novel concepts to emerge [57]. Open coding generated descriptive and conceptual codes by reviewing small segments of the interviews and comparing them to each other. These codes were then refined using axial coding where “code labels and the data linked to them are rethought in terms of similarity and difference” and only the codes that best illustrated concepts and relationships found in the data were kept [57]. The remaining codes that emerged from this process fell under five (5) broad categories: (1) distrust; (2) ecological beliefs; (3) human-leopard conflicts; (4) illegal killings and; (5) solutions. The Table 1 provides an overview of the specific codes and quotations included under them within these broader conceptual categories.

## 3. Results

A significant majority of farmers and NGO staff (9 out of 10) in the study area suggested that local leopard killings are common, and tolerance for this crime is relatively high. Of the ten respondents, only one farmer denied that retaliatory killing took place. Contrary to the interviews with farmers and NGO staff, however, the majority of South African government officials denied that any such killings took place in the area. Leopards in the area were primarily shot or caught via gin traps which are spring traps used to capture an animal’s leg and left to die of dehydration and stress. When a farmer lost livestock to a leopard, he called a community member with hunting dogs to chase the leopard on his property into the open or up a tree where it could be shot. According to one government official, once hunted, farmers buried the evidence in the bush, instead of selling the skins. 

### 3.1. Reasons Why Retaliatory Leopard Killings Take Place

Retaliation for Livestock Predation. When asked what they would consider to be the primary reasons for the killing of leopards, the respondents suggested that these killings by farmers took place in retaliation for livestock predation. Conversations with farmers revealed significant anger at leopards, with several farmers calling leopards “criminal” for attacking their private property when wild prey was plentiful. The general feeling was that livestock losses to other causes, such as tick fever, were acceptable, but there was significantly lower tolerance for leopard predation on livestock. This feeling was exacerbated by the belief that human-wildlife conflict had not been a problem in the area until 20 years ago and so farmers felt that leopards had suddenly invaded what had been pristine livestock farming territory. 

Distrust in the Government. Respondents expressed feelings of anger from the perceived economic hardships imposed by leopards, ongoing land tenure and land-use issues, and the lack of government support. Distrust also emerged as an overarching emotion expressed by farmers and government officials.

Two levels of distrust were apparent: (1) general distrust and (2) human-leopard conflict solution-specific distrust. General distrust between local stakeholders stemmed from historical events in the region. Farmers were angry that they received no support from the South African government (from 1867 to 1947, the mining industry subsidized farming but no longer do). Expanding African markets and government protections turned South Africa into one of the few countries that exported food despite fickle rainfall [58]. South Africa is no longer the farming superpower it once was, especially since its economic decline from the 1960s to the mid-1990s, and its history of extractive policies and overgrazing that destroyed farmlands and their topsoil [58]. Farmers felt that they should continue to receive subsidies to keep their crops and beef competitive on the world market. 

Adding to this general distrust was solution-specific distrust because farmers felt that the government had failed to adequately respond to human-leopard conflict while government officials defended their solutions. Specific complaints about solutions from farmers, government officials, and NGO staff were very similar, although none of the parties recognized this. The general distrust made it impossible for parties to see common ground and exacerbated ongoing tensions over human-leopard conflict solutions proposed in the area, fueling solution-specific distrust.

All respondents’ complaints focused on four main problems: (1) the government’s response was slow and unwilling; (2) the government’s response involved inefficient methods; (3) there were inadequate resources to respond and; (4) there was a clear lack of laws or their application. Table 2 provides more detail on each of these emerging themes. 

Other Reasons. According to the farmers interviewed, additional reasons for illegally killing “problem leopards” were that permits to legally kill them took too long to obtain. Farmers did not want to risk more losses and feared not being able to catch the leopard if they waited. In contrast, NGO staff and government officials specified that farmers sometimes killed leopards out of spite without much proof that the leopards were responsible for the attacks on their livestock. This disconnect in perspective fueled general distrust and solution specific distrust amongst all involved.

### 3.2. Seeking Government Help When Dealing with Human-Wildlife Conflict

Farmers’ Perspectives. Farmers also provided more specific reasons as to why they did not call government officials when they had a problem leopard. For example, farmers complained that government officials used cage traps that were too small, reducing the chances of capturing problem leopards on their property. In the event that a problem leopard was caught in a cage trap, farmers argued that government officials did not relocate the animal far enough to prevent it from coming back to their farm. One farmer asked a veterinarian to tag a leopard trapped by the government on his land. Despite government assurances that they would relocate the leopard too far for it to return, when the farmer trapped “another problem leopard” with government help a few weeks later, the veterinarian confirmed that this “other leopard” was actually the original animal, which had returned to the farm after relocation. Farmers also thought that government officials were feeding leopards beef during translocations and that this habit was causing them to eat livestock upon their release. Whether or not these complaints were legitimate, the interviews made clear that farmers had strong feelings and preferences on the types of human-leopard conflict solutions that should be put in place, and they felt that their views were not being heard.

Government’s Response to Farmers’ Concerns. Despite the disagreement between farmers and government officials and NGO staff about the size of cage traps, during the interviews, government officials did acknowledge broader failures in their responses to human-leopard conflict. Some recognized that the techniques often used, such as trapping or bell collars, were not always effective because they did not consider each farm’s specific terrain or livestock farming practices. Government officials were aware that predation prevention measures were time consuming and burdensome for farmers to implement effectively. They also recognized that the penalties for illegal leopard killings were not a deterrent given the rarity of prosecution and the lack of other easy solutions to human-leopard conflict.

Government officials also acknowledged that they struggled to respond fast enough to human-leopard conflict complaints because of trained personnel shortages and lack of equipment. On the other hand, farmers rarely received monetary compensation from the government and its haphazard response to human-leopard conflict left them feeling like the government was powerless and uninterested in helping. Any help received was often inconsistent, breeding more distrust between the parties. The maelstrom of anger, distrust, and inefficiencies in response created a toxic environment where simple agreement on the incidence of retaliatory killings or even the most effective solutions was impossible.

### 3.3. Solutions Proposed to Deal with Human-Wildlife Conflict Involving Leopards

Respondents were asked to elaborate on the various solutions that were either proposed or that they or others had implemented to deal with human-leopard conflict in the study area. The respondents were also asked if they thought these solutions were effective and, if so, why. The reasons provided by farmers versus those of government officials and NGO staff varied. Despite ongoing distrust between the parties, there was a significant amount of unacknowledged agreement on what solutions did or did not work. These reasons are summarized in Table 3. While all parties expressed the reasons why these solutions did not work in different ways, their arguments had a lot in common even if the distrust between them prevented them from recognizing or accepting their common ground.

For example, when asked about the effectiveness of translocation, farmers argued that translocation was a temporary solution, as translocated leopards quickly returned to their original territories, a finding that has been verified through a study conducted in India [17]. Meanwhile, government officials and NGO staff believed that translocation stressed leopards and sparked inter-leopard conflict over territory, hurting leopard survival. Inter-leopard conflict can drive leopards to return to their original territory, so all respondents, in essence, agreed that translocation could result in leopards returning to their original territory, rendering the solution ineffective. Nevertheless, some government officials and NGO staff wanted leopards trapped and released without proof that fear of humans would keep them away from livestock. Farmers rejected this solution outright. This willingness to disregard evidence of ineffectiveness aggravated ongoing distrust between farmers and government officials and NGO staff. 

Farmers and government officials also agreed that killing a damage-causing leopard was sometimes a viable solution but differed on when to do so (NGO staff did not agree). Farmers preferred to shoot first to avoid further immediate conflict. However, Government officials and NGO staff preferred to try non-lethal methods before killing. All respondents recognized that alternative predation prevention methods, like Anatolian sheep dogs, donkeys, and wire/bell collars, were inconsistently successful and required careful implementation and evaluation. 

There was general agreement on why and when leopard predation and human-leopard conflict occurred. The interviews reveal that farmers and government officials and NGO staff disagreed on who/what was to blame for these conditions: farmers tended to blame poor prevention methods and environmental conditions, while government/NGO staff tended to blame farmers. Given that government officials and NGO staff often advocated for the prevention methods that farmers found ineffective and that farmers felt unsupported and targeted by government officials and NGO staff when they resorted to lethal predation prevention methods, this difference in who was to blame fueled both general and solution specific distrust between the parties. In turn, this distrust made it impossible for parties to see their common perspectives in what were the best solutions to prevent human-leopard conflict creating a vicious cycle of failed responses.

Collectively, farmers conjured up the most innovative ideas to prevent leopard livestock predation including farmer-sponsored insurance schemes and certain product designations for farms that use leopard-friendly predation prevention methods. Farmers also suggested diversifying farming practices to avoid being vulnerable to livestock losses and rotating livestock around to different pastures to avoid repeat predation. Government officials’ main approach was to research other countries’ solutions to human-wildlife conflict, but it appeared more promising to harness farmers’ practical knowledge to find innovative solutions to human-leopard conflict. However, at the time of the interviews, the anger and distrust between farmers, government officials, and NGO staff was preventing such collaboration.

## 4. Discussion and Conclusions

### 4.1. Summary of Findings

This exploratory qualitative research was designed to understand why retaliatory killings occur in a small village in South Africa and how to best prevent them. Through in-depth interviews, this research was able to identify facilitating and mitigating factors for these killings as well as predation prevention methods that have a strong potential to limit retaliatory killings based on empirically tested crime prevention techniques.

Our results showed that farmers made rational choices about when to kill leopards. These choices, triggered by leopard predation on their livestock, were shaped by a number of choice-structuring properties, among which were government’s unwillingness to respond to human-leopard conflict; the government’s use of inefficient methods in situations when it did respond to this conflict; the lack of overall adequate resources to respond and; the clear lack of laws or their application when dealing with the conflict. All local stakeholders in this study had strong opinions about what solutions would or would not work to prevent opportunities for livestock predation and future leopard retaliatory killings. This research uses situational crime prevention as a framework through which to understand how and why these various proposed solutions would prevent and control illegal leopard killings or leopard-livestock predation.

### 4.2. Study Limitations

One important limitation of this research is its small sample size. There were significant challenges associated with locating individuals from the stratified groups who were willing to talk to the researcher, particularly given the remote location of farms. Given the exploratory nature of this research and the methods designed to collect in-depth data on the knowledge and experiences of the respondents, as well as the fact that the researchers did not seek generalizability, it was decided that a sample size of 16 respondents was sufficient. For studies that have small sample sizes, it is important to supplement such findings by citing supporting literature, and the findings from these interviews reflect the existing literature related to human-wildlife conflict [59]. Additionally, a large body of social science research supports the assumption that for qualitative research, a large sample size or interview pool is not always necessary [60,61,62,63]. A significant amount of social science research on “hard to reach” populations has been conducted in the past with small sample sizes despite which the researchers provided valuable insights about the studied phenomena [64,65,66,67]. 

The snowball sampling method for this research is another potential limitation, but snowball sampling was necessary to get such a tight-knit rural community to talk to a foreign researcher [68]. Respondents needed to hear from another community members that talking with the researcher was acceptable and encouraged to open up in interviews. The use of the word “poaching” could also have influenced the respondents’ answers given the sensitive nature of the topic and the fact that this terminology can sometimes be stigmatizing. When speaking with the respondents, the researcher made sure to explain that there was no stigmatization intended and then shifted to whatever terminology the respondent preferred. The amount of sensitive and detailed information given during in-person interviews and the range of opinions expressed, including controversial ones, suggest that most respondents were honest in their responses. The choice to only write down key words during interviews and write-up the content immediately afterwards likewise made respondents more comfortable and allowed for more open communication about their problems with leopards. The downside of this method was possible content error when writing up interviews from memory, but the general points made by the respondents would have been hard to forget and Halcomb et al. note that recording or transcribing interviews is not necessary for quality data collection [69]. Any hesitation on content was noted in the post-interview write-ups and that information was excluded from the analysis.

### 4.3. Recommendations for Retaliatory Leopard Killing Prevention

Our study site had a culture of distrust among actors which impeded conservation goals and made it difficult to acknowledge common ground or agree on solutions to human-leopard conflict and retaliatory killings. This finding is similar to findings related to the lack of trust in the relationship between local communities and wildlife enforcement in Botswana and Uganda [70,71]. Local residents, like the South African farmers interviewed, have innovative and effective ideas for solutions. These solutions are more likely to be accepted and successful because they consider the local lifestyles, locally available resources, mentalities, and physical environment, and are informed by past failures. Addressing the culture of distrust, a key choice-structuring property for illegal leopard killings is essential for protecting leopards because any response to human-leopard conflict and retaliatory killings will require a coordinated effort between local populations, national wildlife management authorities or government officials, NGOs, and species experts [72]. 

Conflict transformation combined with criminological approaches, such as situational crime prevention, can inform effective interventions and address this distrust [73]. While there was significant distrust between farmers, government officials and NGO staff, one of this research’s key findings is that there was a lot of common ground between parties. There were also many innovative ideas on how to best address the problem of leopard livestock predation and retaliatory leopard killings. The solutions the interviewees proposed were often based, albeit inadvertently, on effective crime prevention interventions as defined by situational crime prevention in other contexts (for a review of the effectiveness of these SCP interventions, see [74]). These solutions worked to block the opportunities for either leopards to prey on livestock or for farmers to kill leopards in retaliation for predation. Below, we describe, based on proposed solutions and crime prevention principles, some examples of what should be kept as best practices to prevent both retaliatory leopard killings and its trigger, human-leopard conflict.

When designing prevention-based solutions to these problems, interventions should first focus on preventing leopard predation on livestock. Donkeys protect the herd, are already semi-accepted as a predation prevention method in South Africa, and are cheap to buy. Predator-proof corrals, ideally with wire mesh roofs and fencing at least a foot deep in the ground to prevent leopards from jumping or digging into enclosures, can also be used [75,76]. “Lion lights” with motion sensors could also deflect predators away from livestock corrals [77]. Ideally, these methods are applied in tandem for maximum protection. Reducing the opportunity for committing retaliatory leopard killings can then be done in two ways: (1) making tools less accessible, like banning over-the-counter sales of pesticides used to poison livestock carcasses, and; (2) immediately investigating any cases of missing collared leopards to increase the risk that an offender is caught before the disposal of the body.

Livestock owners can also identify and manage leopard predation risk factors in their environment. A simple cellphone application that predicts likely predation locations (e.g., based on collared leopard movements or previous instances of predation) could help livestock owners decide where to graze their livestock and when to corral them for safety. This could reduce leopard attacks on livestock and people, thus limiting provocations for retaliatory killings. Game scouts can also track leopard movements and warn farmers or guard livestock when leopards are nearby (similar to the Lion Guardians’ model) [78]. Communities should implement predation response teams to immediately address complaints of human-leopard conflict and work with livestock farmers to create predation prevention plans that work for them, as implemented for human-tiger conflict near India’s Corbett Tiger Reserve [79]. These teams should rapidly deploy after a complaint to minimize the risk of retaliation.

Another strategy to reduce human-leopard conflict could be randomly moving livestock around to different grazing areas to avoid repeat predation. Predation response team members could also work with a farmer to reduce tick fever losses, which, according to the respondents, account for far more livestock deaths than leopards, thereby decreasing financial losses [80]. They might also suggest that farmers monitor their flock regularly and immediately dispose of any carcasses to dissuade leopards from returning to a kill site and attacking more livestock.

The government could also consider giving farmers financial incentives to conserve leopards since South African farmers complained of the lack of government subsidies during their interviews. It could consider subsidizing farmers who set aside land for conservation and implement predation prevention techniques, giving farmers an incentive to tolerate some livestock predation. More importantly, communities can and should be empowered to create their own solutions. Two ways to do this would be to have government officials and NGO staff support local leadership for conservation and offering local residents micro-loans to develop their own predation prevention methods.

One important way to remove excuses for retaliatory killings is to make it easy to report them by, for example, creating hotlines and cell phone applications where local residents can report illegal killings anonymously. Wildlife officials also need to establish a positive presence in the community, similar to community policing officers. As an example, the Uganda Wildlife Authority created such a unit to liaise between wildlife enforcement officers and the community [71]. Wildlife officials could stop by every farm, introduce themselves, and learn about farmers’ struggles with human-wildlife conflict, or, alternatively, designate a community member to be a law enforcement liaison [80]. These efforts would reduce the distrust farmers generally have for conservation practitioners and allow for open discussion of ongoing community problems.

Conflict resolution techniques could also reduce distrust and foster cooperation between local residents, government wildlife officials, and NGO staff. For example, the Kenyan Wildlife Service (KWS) has held community outreach meetings with local residents and researchers to discuss the human-wildlife conflict solutions they were implementing [81]. These forums help communities understand KWS’ actions and allow local residents to give feedback on whether or not these solutions are working. Similarly, participatory crime analysis workshops, where residents come together to discuss where crime occurs in their community and why to design tailored solutions, improve relations between people and local law enforcement by empowering local residents to protect their communities from crime [82]. These events can be a place to openly discuss conflicts and showcase common ground between parties, thus building trust [83].

Our research has shown that locally designed and implemented solutions exist in the study site and that these solutions address the essential components for effective prevention of human-wildlife conflict and retaliatory leopard killings through key situational crime prevention techniques. These techniques focus on dissuading illegal behavior, negating its reward, or neutralizing triggers for it. What they do not currently do is consider positive incentives for behavior change nor specifically highlight the role of informal guardians in crime prevention. This gap was apparent during our research when respondents highlighted solutions like revenue sharing from tourism that encourage farmers to conserve leopards by giving them positive value. Another such example was the point person with leopard hunting dogs that farmers called when they experienced a predation event, an informal guardian for livestock that farmers set up themselves where formal government efforts failed. These gaps suggest the need for adapting crime prevention frameworks to better fit new disciplines in which they are applied, like conservation. We encourage researchers and practitioners to work together to do so (we provide one such adaptation based on this research in Table A1 in Appendix A; others also exist [53,84,85]). Environmental criminology has a lot to offer conservation when it comes to tackling crime problems like illegal leopard killings, but only when disciplines work together and with local communities to identify what works and why, will crime prevention tools be effectively leveraged to solve conservation problems. The views expressed herein are those of the author(s) and do not necessarily reflect the views of the United Nations.

## Figures and Tables

**Table 1:** List of Codes Generated During the Content Analysis in Atlas.ti.

Code Name	Applied When…
**Distrust**	
Anger	Expressions of anger at leopards, the current state of predation, the current conservation situation, or actors involved in the area.
Fear	Quotations suggesting that local actors live in fear as a result of human-wildlife conflict.
Distrust	Quotations suggesting that stakeholders do not trust either other stakeholders in the study site or members of their own group.
Corruption	Any reference to corruption as a factor in how leopard livestock predation and illegal leopard killings are investigated or dealt with in the area. Quote must include the word “corruption.”
Failure of government response	Comments suggesting the government’s current handling of human-leopard conflicts and illegal leopard killings is not working.
**Ecological Beliefs**	
Rationale for predator intolerance	Quotes that give reasons why a person dislikes leopards.
Livestock loss expectation	Quotes describing farmers’ beliefs about acceptable losses of livestock. Quote must include the words “livestock loss” or any of its derivatives (i.e., “lost livestock”).
Natural prey availability	Quotes describing farmers’ beliefs about how much natural prey is available in the area for leopards to eat.
Unsustainable land use	Quotes describing unsustainable land uses or practices in the area.
**Human-Leopard Conflict**	
Human-wildlife conflict cost	Examples of how human-wildlife conflict imposes a cost on humans and/or wildlife.
Predation prevention beliefs	Quotes explaining what methods biologists, NGOs, government officials, farmers, and the general public believe should be used to prevent leopard livestock predation.
Why & when predation	Quotes describing why and when biologists, NGOs, government officials, farmers, and the general public believe leopards eat livestock.
**Illegal Killings**	
Rationale for leopard killing	Justifications individuals provided for why they believe it is acceptable to shoot a leopard.
Poaching network	Quotes suggesting that offenders had help or belong to an organized group.
Leopard killing method ^1^	Quotations describing how leopards are killed in the area.
Why & when killed	Quotes describing why and when biologists, NGOs, government officials, farmers, and the general public believe leopard poaching and retaliatory killings take place.
**Solutions**	
Judicial outcome	Description of judicial outcomes for prosecutions of leopard killings/poaching.
Solutions: Suggested & attempted	Description of any suggested or attempted solutions to prevent leopard livestock predation and illegal leopard killings.
Solutions: Why working	Quotes that explain or suggest why certain solutions to poaching and retaliatory killings are working.

^1^ All interviews were also coded on whether or not respondents believed leopards were being illegally killed in the region.

**Table 2:** Respondents’ complaints about the official response to leopard predation and retaliatory killings.

Types of Complaints	Farmers’ Complaints	Government Officials’ and NGO Staff’s Complaints
Using inefficient methods	Officials feed meat to leopards during translocation which encourages livestock predation	Officials sometimes lack training and use ineffective methods
	Officials do not relocate far enough	Officials use “one size fits all” enforcement or simply do not have resources to tailor solutions to farmer needs
	Official trapping cages are too small allowing leopards to escape	Prevention measures are just one more burden for farmers, so unlikely to stick to them
		Penalties are not a deterrent for farmers who kill leopards
Inadequate laws	Officials apply laws unequally	No national legislation for these issues makes enforcement difficult
Not enough resources	Government authorities provide no compensation for predation losses	Officials lack enough money to do things properly
		Officials do not have enough trained personnel to respond
Slow & unwilling	Officials are corrupt	Officials wait too long to act
		Corruption is an issue
	Officials are uninterested in solving the problem	No one will talk to help investigators and/or wildlife management personnel

**Table 3:** Responses to human-leopard conflict cited by respondents and their perceived effectiveness.

Predation Prevention Methods Cited	Farmers’ Thoughts on These Methods	Government Officials’ and NGO Staff’s Thoughts on These Methods
Translocation	Ineffective because leopard comes back	Stress of translocation hurts leopard survival
Kill problem leopard	Best to kill leopard right away	Only if trapping ineffective, issue kill permit
	Use poison collars on livestock if needed	
Trap & release	Completely ineffective	Trap & release with GPS, hope for fear of humans, no guarantees
Anatolian sheep dogs	Dogs cause other problems by hunting small prey	Work when hunting instinct bred out of dogs
Donkeys	Ok, but not 100% effective	Work well
Wire collars	Ineffective	Ineffective
Bell collars	Ineffective	Good when used randomly so leopards are not habituated to the noise
New ideas	Suggested alternative solutions: Keep horns on meat cattle so they can protect themselves from leopards Create a leopard predation compensation fund that farmers pay into Move livestock to different pastures regularly to avoid repeat predation Create leopard-friendly products label so farmers can charge higher prices when using predation prevention methods, encouraging their use	Suggested alternative solutions: Work on a case-by-case basis with farmers to find tailored solutions Build predator-proof enclosures to protect livestock

## Data Availability

The data will be available with the corresponding author upon request.

## Appendix A

**Table A1:** Modification of the 25 techniques of situational crime prevention for illegal leopard killings in the South African context *^,1^.

Increase Effort	Increase Risks	Reduce Rewards	Reduce Provocations	Remove Excuses
Harden targets	*Recruit informal* *guardians* *e.g., community member with dogs called after predation events*	Conceal targets	Reduce frustrations	Set rules
Control access	Assist natural surveillance	*Encourage ownership* (*of wildlife*) *to increase agency for wildlife* *protection among farmers*	*Increase transparency* *among government institutions to limit corruption or the perception of it among farmers*	Post instructions
Screen exits	Reduce anonymity	Disrupt markets	Avoid disputes	Alert conscience
Deflect offenders	Place managers	Deny benefits	Reduce emotion	*Cooperative extension* *e.g., education initiatives to encourage cooperation from communities to prevent illegal killings*
*Control tools (incl.* *drugs & alcohol)*	Strengthen formal surveillance		*Harness peer pressure* *by having respected community members positively pressure farmers to protect rather than kill leopards*	*Increase incentives &* *alternative benefits* *e.g., tourism revenue sharing with farms that use leopard friendly predation prevention methods*
*Leverage handlers* *e.g., pro-conservation farmers encouraging peers to protect leopards*			Discourage imitation	

* The modified techniques are italicized with illustrative examples below them. The original 25 techniques of situation crime prevention can be found here for reference: https://popcenter.asu.edu/sites/default/files/twenty_five_techniques_of_situational_prevention.pdf (accessed on 13 September 2021). ^1^ Originally presented at the Environmental Criminology and Crime Analysis conference: Kahler, J.S., Viollaz, J.S., & Gore, M.L. (2018). Beyond the urban crime jungle: Refining the situational crime prevention framework for wildlife crime. ECCA: Elche, Spain.

## Appendix B

Interview questions for farmers with knowledge (first or secondhand) of poaching.

(1) Why do you think poaching occurs?
  1. What factors make it easier/possible for poachers to operate?
(2) What do local residents think about poaching?
  1. Are they for it or against it?
  2. Do they see the ills or just don’t care much because the consequences of poaching do not directly affect them?
  3. Do they view the land and its resources as theirs to use as they please?
(3) Do you know of anyone who poaches? If so, who are they (no names just background information on the person)?
  1. How many are there?
  2. Do they work in groups (organizational structure loose or strictly defined)?
  3. What are their financial circumstances?
  4. Do they have any specific knowledge or experience with wildlife?
(4) Why do these people poach? What incentives do these individuals respond to (with an eye to using that to find an alternative to poaching)?
(5) What negative effects have you personally felt in your community from poaching?
  1. Are there any indirect effects from poaching that you’ve noticed and that wouldn’t be evident to the casual observer?
  2. Do most people in your community experience any negative effects from poaching?
  3. Is this a topic that comes up among community members?
(6) Do you know where poachers unload their products (identify markets)?
  1. Who has access to these markets?
  2. Ask if possible to see such a market.
(7) What do you believe would be the best methods to combat poaching in your area?
  1. What would it take to implement these methods?
  2. Who should be responsible for these efforts?
(8) What obstacles currently exist to effectively combating poaching in your area?
  1. Are there any issues of corruption, cultural practices, lack of resources, or lack of interest from authorities?
  2. Are people in your area interested in helping stop poaching?
  3. What perception do community members have of current anti-poaching efforts or of wildlife management personnel in general?
(9) Have there been any previous efforts made to stop poaching in your community?
  1. Were they successful?
  2. What made them successful/unsuccessful?
  3. What would you have done differently than they did?
  4. Are they still being implemented? If not, why not?
